# Synthesis, Characterization and Dye Removal Behavior of Core–Shell–Shell Fe_3_O_4_/Ag/Polyoxometalates Ternary Nanocomposites

**DOI:** 10.3390/nano9091255

**Published:** 2019-09-04

**Authors:** Shixia Zhan, Chunyan Li, Heyun Tian, Chenguang Ma, Hongling Liu, Jie Luo, Mingxue Li

**Affiliations:** Henan Key Laboratory of Polyoxometalates, Institute of Molecular and Crystal Engineering, College of Chemistry and Chemical Engineering, Henan University, Kaifeng 475004, China

**Keywords:** ternary nanocomposites, magnetic–opto properties, dye removal

## Abstract

The ternary nanocomposites Fe_3_O_4_/Ag/polyoxometalates (Fe_3_O_4_/Ag/POMs) with core–shell–core nanostructure were synthesized by coating [Cu(C_6_H_6_N_2_O)_2_(H_2_O)]H_2_[Cu(C_6_H_6_N_2_O)_2_(P_2_Mo_5_O_23_)]·4H_2_O polyoxometalates on the surface of Fe_3_O_4_/Ag (core–shell) nanoparticles. The transmission electron microscopy/high resolution transmission electron microscopy (HR-TEM) and X-ray powder diffraction (XRD) analyses show that the Fe_3_O_4_/Ag/POMs ternary nanocomposites reveal a core–shell–core nanostructure, good dispersibility, and high crystallinity. The vibrating sample magnetometer (VSM) and physical property measurement system (PPMS) demonstrated the good magnetic properties and superparamagnetic behavior of the nanocomposites at 300 K. The UV–vis spectroscopy displayed the broadband absorption of the Fe_3_O_4_/Ag/POMs with the maximum surface plasmon resonance of Ag nanostructure around 420 nm. The dye removal capacity of Fe_3_O_4_/Ag/POMs was investigated using methylene blue (MB) as a probe. Through adsorption and photocatalysis, the nanocomposites could quickly remove MB with a removal efficiency of 98.7% under the irradiation of visible light at room temperature. The removal efficiency was still as high as 97.5% even after six runs by magnetic separation of photocatalytic adsorbents after processing, indicating the reusability and high stability of the nanocomposites. These Fe_3_O_4_/Ag/POMs photocatalytic adsorbents with magnetic properties will hopefully become a functional material for wastewater treatment in the future.

## 1. Introduction

Multifunctional nanocomposites of different compositions have greater attractiveness in basic research and application compared with their one-component counterparts due to their distinct dimensional structure, rich composition, and better performance [1,2,3,4,5]. The compositions are abundant and versatile, including magnetic, semiconductor, organic, inorganic, and metallic materials [6,7,8,9,10]. Magnetite (Fe_3_O_4_) and noble metal silver (Ag) are two very important typical materials used in a large number of studies on magnetic and metallic materials. Fe_3_O_4_ holds an extraordinary position in the field of magnetic materials, arising from not only its unusual physicochemical properties [11,12] but also from its non-toxic and excellent biocompatibility, which is especially interesting for applications for the biomedical community such as drug delivery, cancer treatment, biosensor, magnetic resonance imaging, etc [13,14,15,16]. Simultaneously, Fe_3_O_4_ also possesses a number of interesting phenomena, like mixed valence, charge ordering, superparamagnetic behavior, and metal-insulator transitions called Verwey transitions [11,17]. Also, Fe_3_O_4_ has a large constant magnetic moment and can be easily collected by using an external magnetic field placed outside the extraction container without additional centrifugation or filtering of the sample, making sampling and collection easier and faster [18,19,20,21,22]. Alternatively, Ag has unparalleled advantages in chemical stability, flexibility, biological activity, and selectivity as an environmentally friendly material, and is widely used in biology, catalysis, medicine, and other fields [23,24,25,26,27]. Moreover, Ag nanoparticles have strong surface plasmon resonance (SPR) absorption and high electron capture performance in the visible region [28,29], which can be used to modify the visible light absorption and carrier generation and separation of materials, as well as enhance the photocatalytic activity of the materials. At the same time, Ag also provides a new electron transport pathway for charge carriers and can enhance conductivity [30]. Combining these two materials by synthesis into a single nano-entity has been successfully achieved with exciting results, such as particle size controllable or different core-shell ratios of nanoparticles [31,32,33].

Furthermore, polyoxometalates (POMs), as new types of porous material with rich composition, high negative charge and various geometric topologies, have been widely used as adsorbents for removing inorganic and organic contaminants [34,35,36,37]. Unfortunately, most POMs are readily soluble [38]. In order to solve the recycling problem of POMs, POMs are often combined with nanomaterials to improve the utilization of POMs.

Now, a large amount of organic dye wastewater discharge poses a major threat to the aquatic environment and human health due to its toxicity and carcinogenicity [39]. There is an urgent need to find an ideal remover that not only reduces the organic dyes in the contaminants with high efficiency and low loss, but also achieves selective separation and recovery to achieve a green chemistry concept. Nanocomposites have attracted great interest due to their versatility. We previously synthesized Fe_3_O_4_/POMs nanocomposites processing excellent adsorbability for cationic dyes. The magnetic Fe_3_O_4_/POMs nanocomposites are beneficial for the separation of adsorbents after processing [40]. In this work, Fe_3_O_4_, Ag, and POMs three materials are combined into a single entity by nanoengineering, the final nanostructure performing the unique properties of Fe_3_O_4_, Ag, and POMs, simultaneously, would bring out novel collective phenomena due to the interaction of Fe_3_O_4_, Ag, and POMs. The spherical ternary nanocomposites were synthesized first by encapsulating Ag on the surface of Fe_3_O_4_ core to form core–shell Fe_3_O_4_/Ag nanoparticles, and then coating the Fe_3_O_4_/Ag nanoparticles with POMs to form core–shell–core Fe_3_O_4_/Ag/POMs nanoparticles. The research on their magnetism revealed that the nanocomposites exhibited superparamagnetic behavior. By applying an external magnetic field, the nanocomposites could be easily separated from the mixed solution and reused. In the methylene blue (MB) removal experiment, the nanocomposites not only exhibited good adsorption performance, but also exhibited increased photocatalytic performance and could more completely remove MB. In the cycle test, the removal efficiency of MB by nanocomposites was almost similar, indicating the reusability and high stability of the nanocomposites. The high efficiency and cyclability of the nanocomposites in dye removal can be attributed to the synergistic effect of the three components in the nanocomposites.

## 2. Experimental

### 2.1. Materials and Measurements

All chemicals were obtained from Aldrich (Beijing, China). The Fe_3_O_4_/Ag nanoparticles were synthesized by Iron(Ⅲ) acetylacetonate (Fe(acac)_3_, 99.9%, Aldrich), Ag acetylacetonate (Ag(acac), 99.9%), poly(ethylene glycol)–block–poly(propylene glycol)–block–poly(ethylene glycol) (PEO–PPO–PEO, Mn = 5800), octyl ether (C_8_H_17_OC_8_H_17_, 99%), and 1,2-hexadecanediol (C_14_H_29_CH(OH)CH_2_(OH), 90%). Polyoxometalates (POMs) was synthesized by copper(II) perchlorate hexahydrate (Cu(ClO_4_)_2_·6H_2_O, 98%), sodium molybdate dihydrate (Na_2_MoO_4_·2H_2_O, 99%), pyridine-2-carboxamide (C_6_H_6_N_2_O, 98%), and phosphoric acid (H_3_PO_4_, 85%). During the experiment, all materials were processed without further treatment, and distilled water was used for the experimental water.

The structures and morphology of Fe_3_O_4_/Ag/POMs nanocomposites were analyzed by XRD (X’Pert Pro, Bruker, Karlsruhe, Germany) using Cu Kα radiation in the angular range 2*θ* = 5°–90° at 293 K and TEM (JEOL 2010F, JEOL Ltd., Tokyo, Japan). The elements of Fe_3_O_4_/Ag/POMs nanocomposites were analyzed using X-ray photoelectron spectrometer (XPS, Thermo Scientific Escalab 250Xi, Waltham, MA, USA) with Al Ka X-ray as the excitation source. The UV–vis spectroscopy was acquired from a TU-1900 spectrometer at room temperature (UV–vis, HitachiU4100, Beijing Purkinje General Instrument Co., Ltd., Beijing, China). The Fourier transform infrared spectroscopy (FTIR, Nicolet Company, Waltham, MA, USA) studies of POM, Fe_3_O_4_/Ag nanoparticles and Fe_3_O_4_/Ag/POMs nanocomposites were recorded in the wavelength range of 500–4000 cm^−1^. The dried samples were magnetically measured using VSM (Lakeshore 7300, Westerville, OH, USA) and PPMS (Quantum Design, San Diego, CA, USA) to evaluate the magnetic properties of the nanocomposites in relation to the applied magnetic field and temperature.

### 2.2. Synthesis of Fe_3_O_4_/Ag

The Fe_3_O_4_/Ag nanoparticles were prepared in the order of synthesis of Ag on the surface of the Fe_3_O_4_ seeds similar to that reported in the literature [41]. First, the Fe_3_O_4_ seeds were produced in the presence of Fe(acac)_3_ (0.1324 g, 0.375 mmol), 1,2-hexadecanediol (0.6461 g, 2.5 mmol), PEO–PPO–PEO (0.7878 g) and octyl ether (10 mL) at a high-temperature (280 °C for 1 h). Then, after cooling down to room temperature, Ag(acac) (0.0258 g, 0.125 mmol) with 2 mL octyl ether was added to the solution to coat with Ag nanolayers. In the process, the reaction mixture was heated to 80 °C in 1 h and the temperature kept for 2 h, then rapidly heated to 215 °C and refluxed for 2 h. After the reaction was complete, the light brown powder was obtained by centrifuging, washing with ethanol–hexane (1:2), and drying at room temperature. The Ag element content was 44.21% and the Fe element content 40.13% in the Fe_3_O_4_/Ag nanoparticles (Figure S1).

### 2.3. Synthesis of POMs

The POMs were synthesized using the method reported in the literature [40,42,43]: Taking Cu(ClO_4_)_2_·6H_2_O (0.093 g, 0.25 mmol) and pyridine-2-carboxamide (0.061 g, 0.5 mmol) in a beaker, and adding 15 mL of distilled water to prepare a solution, and stirring at 50 °C for 0.5 h. After cooling to room temperature, 10 mL aqueous solution of Na_2_MoO_4_·2H_2_O (0.242 g, 1.0 mmol) was added to the solution, the pH was maintained at 3 by dropwise addition of concentrated H_3_PO_4_, and then the mixture was stirred for 0.5 h at room temperature and filtered. The blue precipitate obtained by filtration was dried by heating to obtain POMs. After filtering the mixture, the powders were stored for the following synthesis. The filtrate was allowed to evaporate at room temperature. After 3 days blue crystals suitable for X-ray studies were filtered off, washed with distilled water and dried in a desiccator at room temperature to give a yield of 38.4% based on Mo. Anal calc. for C_24_H_34_Cu_2_Mo_5_N_8_O_32_P_2_ (1615.30): C, 17.85; H, 2.12; N, 6.94; Cu, 7.87; Mo, 29.70. Found: C, 17.85; H, 2.14; N, 6.92; Cu, 7.88; Mo, 29.66.

### 2.4. Synthesis of Fe_3_O_4_/Ag/POMs

Fe_3_O_4_/Ag/POMs ternary nanocomposites were synthesized via a sonochemical method. In a typical synthesis, Fe_3_O_4_/Ag nanoparticles (5 mg), and POMs powders (50 mg) were added to a 50 mL beaker containing ethanol (10 mL) and water (10 mL), then ultrasonically treated for 10 h to obtain a uniform muddy liquid. After the reaction was completed, the obtained products were gathered using a placed magnet. The magnetic products Fe_3_O_4_/Ag/POMs were washed with ethanol several times. The weight ratio of POMs in Fe_3_O_4_/Ag/POMs was 83.5% (Figure S2).

### 2.5. Dye Removal Experiment

The removal performance of nanocomposites was investigated by measuring the removal efficiency of MB from the solution at room temperature. The concentration of 15 mg/L MB solution was prepared by dissolving the dye powder in distilled water. The dye removal experiment was started with 5 mg of Fe_3_O_4_/Ag/POMs nanocomposites which were dispersed in 30 mL of 15 mg/L MB solution, stirring the mixture in the dark to establish adsorption–desorption equilibrium between the MB molecules and the surface of the remover, following by illumination. The photocatalytic reaction was carried out at room temperature, under simulated visible light provided by a CEL-HXF300 Xe lamp (300 W, Zhongjiao Jinyuan Technology Co., Ltd., Beijing, China) at a distance of 10 cm from the lamp to the reaction beaker. The lamp was equipped with a filter to match the visible light with the wavelength ranging from 400 to 800 nm. About 5 mL of the solution was taken every 2 min and centrifuged at 4500 rpm for 3 min. The filtrate was then analyzed using a UV–vis spectrophotometer to measure the absorption of MB.

## 3. Results and Discussion

### 3.1. Morphology and Monodispersity of Fe_3_O_4_/Ag/POMs Nanocomposites

**Figure 1:**
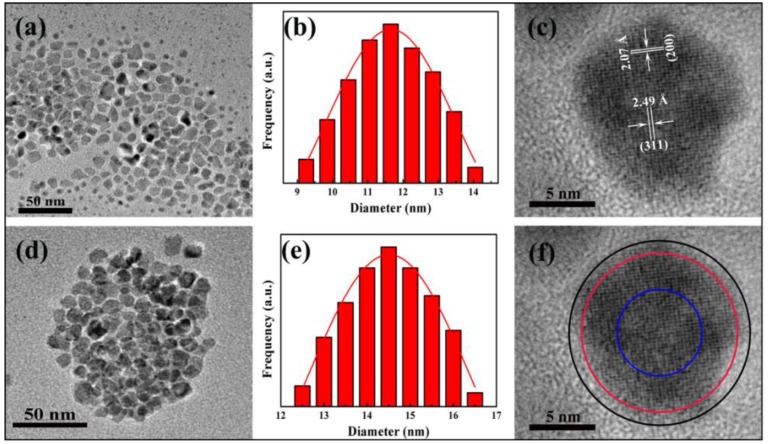
Transmission electron microscopy (TEM) bright-field micrographs of (**a**) Fe_3_O_4_/Ag nanoparticles and (**d**) Fe_3_O_4_/Ag/POMs nanocomposites; particle size histogram with Gaussian fit of (**b**) Fe_3_O_4_/Ag nanoparticles and (**e**) Fe_3_O_4_/Ag/POMs nanocomposites; high resolution transmission electron microscopy (HR-TEM) of an individual Fe_3_O_4_/Ag/POMs (**c**,**f**).

The microstructure and particle size of Fe_3_O_4_/Ag/POMs nanocomposites were analyzed by TEM/HRTEM comparing with Fe_3_O_4_/Ag nanoparticles. As shown in Figure 1a,d, Fe_3_O_4_/Ag/POMs and Fe_3_O_4_/Ag are highly crystalline, substantially spherical and have uniform particle size. The narrow size distributions from size counting of a range of TEM photos can be intuitionistically described by the curve of Gaussian function, and the histograms show that the average size of Fe_3_O_4_/Ag/POMs and Fe_3_O_4_/Ag are ~14.5 nm and ~11.7 nm respectively (Figure 1b,e). The highly regular lattices of single Fe_3_O_4_/Ag/POMs are shown in Figure 1c. As labeled, the lattice spacing of 2.49 Å corresponds to the (311) crystal plane of Fe_3_O_4_, and the spacing of 2.07 Å corresponds to the (200) crystal plane of Ag, which proves that Fe_3_O_4_ and Ag exist simultaneously in the same nanocomposite. The discernible core–shell–core nanostructure can be observed in Figure 1f, in which the shallow center region corresponds to Fe_3_O_4_, the darker shell portions correspond to the heavier element Ag, and the obvious interface lines delimitate the regions of the nanocomposite. These results demonstrate that POMs were successfully covered on the surface of Fe_3_O_4_/Ag, which were in conformity with the further XRD measurements and the aggregation and dispersion experiments. Furthermore, the elemental mappings (Figure S3a–i) illustrate the distribution of the elements Ag, Fe, Mo, P, Cu, C, O, and N in the Fe_3_O_4_/Ag/POMs nanocomposites, indicating that Ag, Fe_3_O_4,_ and POMs coexist in Fe_3_O_4_/Ag/POMs nanocomposites. These further confirm the successful formation of Fe_3_O_4_/Ag/POMs nanocomposites.

### 3.2. XPS Spectra

**Figure 2:**
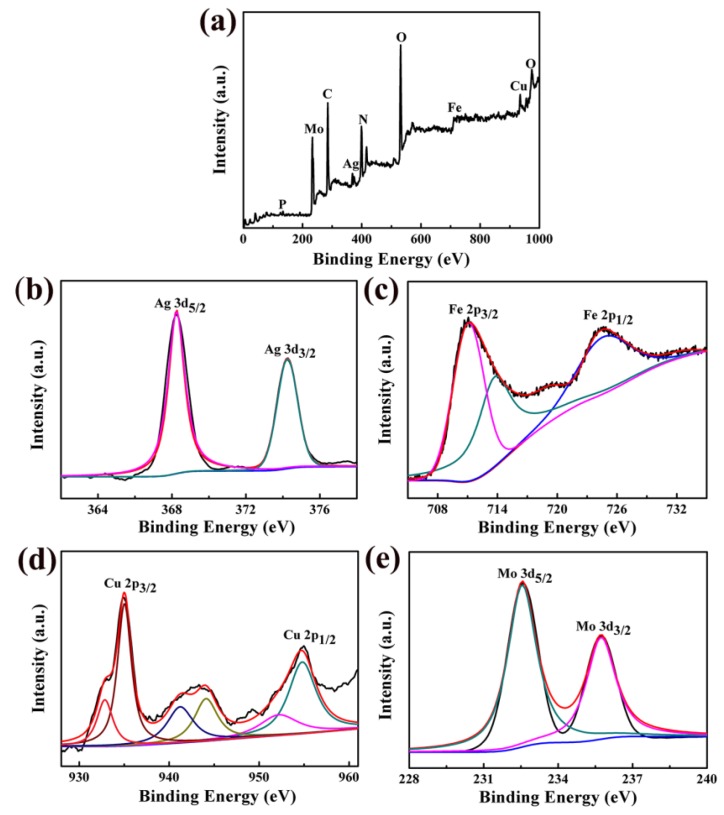
X-ray photoelectron spectrometer (XPS) spectra of Fe_3_O_4_/Ag/POMs nanocomposites: (**a**) full spectrum, (**b**) Ag 3d map, (**c**) Fe 2p map, (**d**) Cu 2p map, (**e**) Mo 3d map.

The XPS study demonstrates the information on the chemical composition of the prepared Fe_3_O_4_/Ag/POMs nanocomposites, and the relevant experiment results are shown in Figure 2. The energy levels of P, Mo, C, Ag, N, O, Fe, and Cu, which were obtained from the full XPS spectra of Figure 2a, are in good accordance with previous reports, confirming the existence of related elements of the nanocomposites. The binding energy of Ag 3d, Fe 2p, Cu 2p, and Mo 3d was calibrated using the C1s peak (BE = 285.7 eV) as a standard. The peaks of binding energy at 368.2 eV and 374.2 eV in Figure 2b correspond to Ag 3d_5/2_ and Ag 3d_3/2_ of pure silver, which proves the existence of Ag [44]. The Fe 2p peaks are resolved into two peaks around 709.1 and 725.0 eV in Figure 2c, which are attributed to Fe 2p_3/2_ and Fe 2p_1/2_ respectively. The binding energy around 709 and 714 eV conforms to Fe^2+^ 2p_3/2_ and Fe^3+^ 2p_3/2_ [45,46]. It could be observed from Figure 2d that the distinct peaks at 935.4 eV and 955.3 eV can be attributed to Cu 2p_3/2_ and Cu 2p_1/2_, respectively, showing an oxidation state of +2. The satellite peaks between Cu 2p_3/2_ and Cu 2p_1/2_ (941–945 eV) provide evidence of the existence of CuO [47]. The peaks observed at 232.6 eV and 235.8 eV (Figure 2e) can be attributed to the Mo 3d_5/2_ and Mo 3d_3/2_, respectively, indicating that the valence of Mo in POMs is mainly VI [48]. The XPS spectra of Cu 2p and Mo 3d are in full compliance with the original skeleton of the POMs. The above analysis proves that Fe_3_O_4_/Ag/POMs nanocomposites contain three components of Ag, Fe_3_O_4,_ and POMs.

### 3.3. XRD Patterns

**Figure 3:**
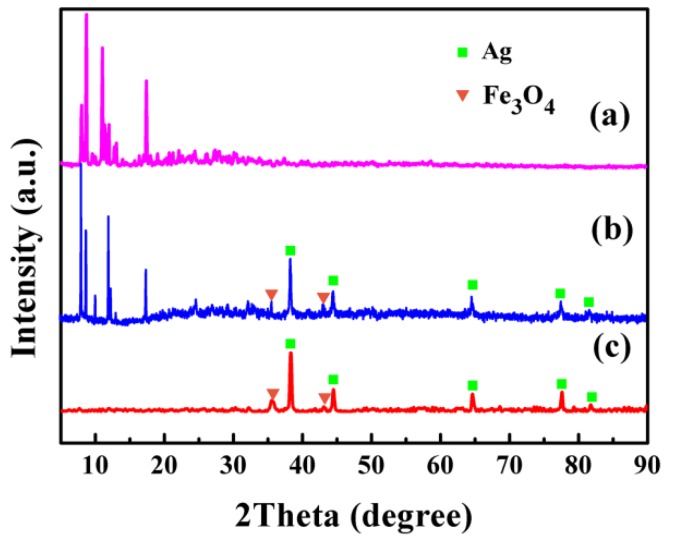
X-ray powder diffraction (XRD) patterns for (**a**) POMs, (**b**) Fe_3_O_4_/Ag/POMs nanocomposites and (**c**) Fe_3_O_4_/Ag nanoparticles (The diffraction peak of Ag is represented by squares, and the Fe_3_O_4_ is represented by inverted triangles).

The crystal structure and the phase purity of the Fe_3_O_4_/Ag/POMs nanocomposites were analyzed by XRD and compared with those of Fe_3_O_4_/Ag and POMs. Figure 3c represents the diffraction pattern of the Fe_3_O_4_/Ag nanoparticles. The diffraction peaks discovered at 38.3°, 44.4°, 64.6°, 77.59°, and 81.75° can be indexed to (111), (200), (220), (311), and (222) planes of Ag (JCPDS No. 87-0720), respectively. The diffraction peaks located at 35.5° and 43.1° are assigned to the (311) and (440) of the Fe_3_O_4_ (JCPDS No. 85-1436). Compared with Fe_3_O_4_, the relatively strong reflection intensity of Ag is mainly attributed to the fact that Ag has a much stronger scattering ability than Fe_3_O_4_ because Ag has a higher atomic number [49]. These characteristic peaks of Fe_3_O_4_/Ag reappear simultaneously in the diffraction pattern of Fe_3_O_4_/Ag/POMs (Figure 3b) together with those of POMs, confirming the formation of Fe_3_O_4_/Ag/POMs nanocomposites.

### 3.4. FTIR Spectroscopy

**Figure 4:**
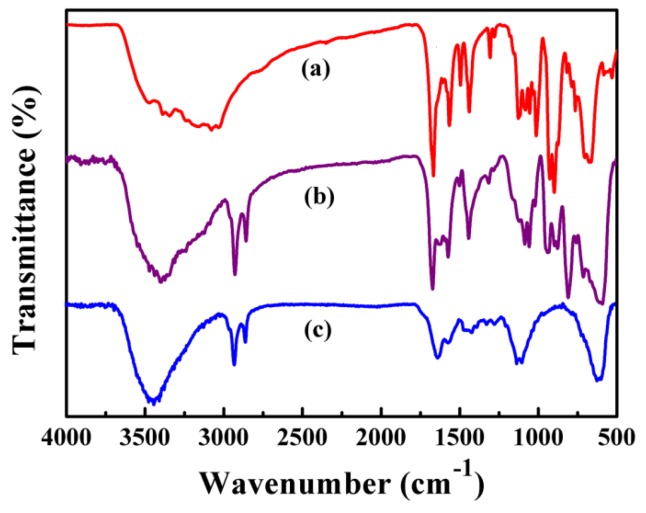
FTIR spectra of (**a**) POMs, (**b**) Fe_3_O_4_/Ag/POMs nanocomposites, and (**c**) Fe_3_O_4_/Ag nanoparticles.

Figure 4 compares the FTIR spectra of POMs, Fe_3_O_4_/Ag/POMs and Fe_3_O_4_/Ag. The IR spectrum of POMs is described in Figure 4a. It is obvious that the characteristic peaks at ~3380 cm^−1^ belong to the O–H stretching vibration of water, and the peak at ~3069 cm^−1^ is related to *ν*(N–H) of C_6_H_6_N_2_O. The series of strong bands are also related to C_6_H_6_N_2_O which are in the range of 1684–1133 cm^−1^. Bands between 1120 cm^−1^ and 1009 cm^−1^ are attributed to *ν*(P–O) stretching vibration [50,51], and the bands at ~906 cm^−1^ and ~669 cm^−1^ are credited to *ν*(Mo = Od) and *ν* (Mo–O–Mo), respectively [51]. The FTIR pattern of Fe_3_O_4_/Ag has a characteristic peak at 584 cm^−1^ owing to the Fe–O skeletal vibrations (Figure 4c) [52]. These characteristic vibrations and bending modes reappear in the FTIR spectrum of the Fe_3_O_4_/Ag/POMs (Figure 4b), further demonstrating that the nanocomposites had been successfully synthesized.

### 3.5. UV–Vis Spectroscopy

**Figure 5:**
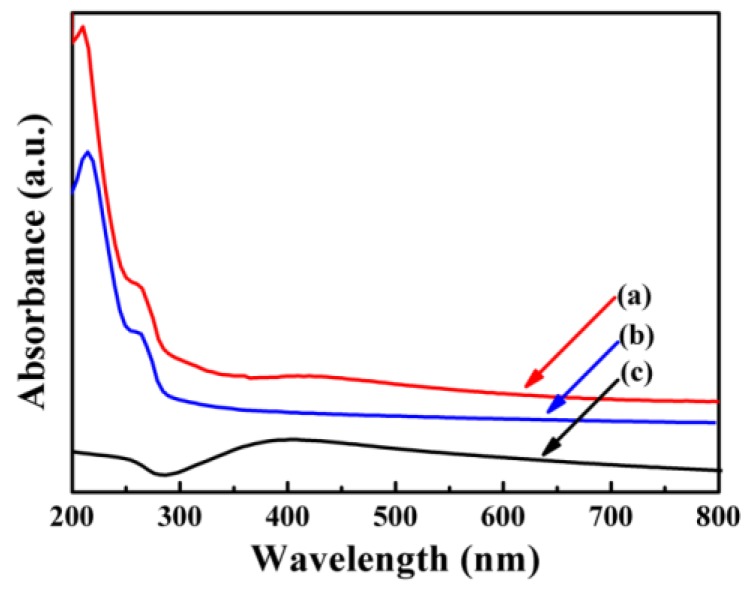
UV–vis absorbance spectra of (**a**) Fe_3_O_4_/Ag/POMs nanocomposites, (**b**) POMs and (**c**) Fe_3_O_4_/Ag nanoparticles dispersed in H_2_O.

The optical property of Fe_3_O_4_/Ag/POMs was assessed by comparing the UV–vis absorption spectra of Fe_3_O_4_/Ag/POMs, POMs and Fe_3_O_4_/Ag dispersed in H_2_O. From Figure 5b, the two absorption bands, which correspond to the charge transfer of Ot→Mo and Ob→Mo in POMs, are noticed near ~210 nm and ~264 nm, respectively [53]. As given in Figure 5c, Fe_3_O_4_/Ag nanoparticles exhibit absorption bands at about 415 nm, which are attributable to the enhanced light trapping of surface plasmon resonance of the Ag nanostructure [54]. As anticipated, in Figure 5a, Fe_3_O_4_/Ag/POMs show absorption bands at about 215 nm and 265 nm arising from POMs, in addition, an absorption band at about 420 nm from Fe_3_O_4_/Ag. The band shape of Fe_3_O_4_/Ag/POMs nanocomposites is similar to that of POMs and Fe_3_O_4_/Ag. The slight shift of the position may be due to the interaction of Ag, Fe_3_O_4,_ and POMs.

### 3.6. Magnetic Properties of Fe_3_O_4_/Ag/POMs Nanocomposites

**Figure 6:**
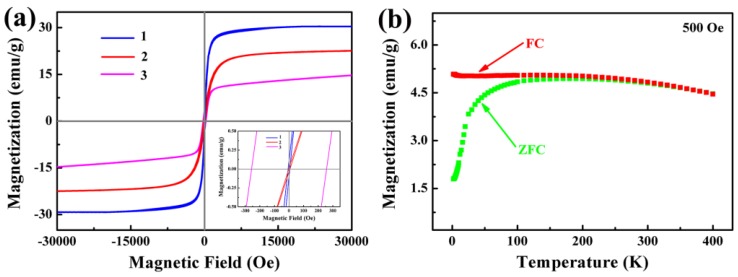
(**a**) Hysteresis curves recorded of Fe_3_O_4_/Ag nanoparticles at 300 K (curve 1), Fe_3_O_4_/Ag/POMs nanocomposites at 300 K (curve 2) and 5 K (curve 3) (Inset: Field response around the origin), and (**b**) ZFC-FC curves of Fe_3_O_4_/Ag/POMs nanocomposites under a magnetic field of 500 Oe.

The magnetic property of Fe_3_O_4_/Ag/POMs nanocomposites was studied by VSM and PPMS. It can be seen from the hysteresis curves in Figure 6a, at 300 K, the coercivity of Fe_3_O_4_/Ag nanoparticles is ~2.19 Oe and the magnetization is ~29.75 emu/g (curve 1), while Fe_3_O_4_/Ag/POMs show a coercivity of ~5.07 Oe and magnetization of ~22.41 emu/g (curve 2). The magnetization decreases due to the POMs coated on the surface of the Fe_3_O_4_/Ag nanoparticles. The Fe_3_O_4_/Ag/POMs nanocomposites possess superparamagnetic behavior at room temperature. The special feature of superparamagnetic nanocomposites is their prompt response to a remote magnetic force. This is because superparamagnetic nanocomposites do not retain magnetism after removal of the external magnetic field. Also, they have large constant magnetic moments and can be easily collected by using an external magnetic field placed outside of the extraction container without additional centrifugation or filtration of the sample [55,56,57]. At 5 K, the coercivity of the Fe_3_O_4_/Ag/POMs nanocomposites increases to 258.24 Oe and the magnetization reduces to 14.21 emu/g (curve 3), indicating that the superparamagnetism is converted to ferromagnetism. Figure 6b shows the magnetization temperature (MT) curves in a field cooling (FC) and zero field cooling (ZFC) mode for a given magnetic field of 500 Oe and a temperature range of 2–400 K. As exhibited in Figure 6b, the blocking temperature (*TB*) is about 220 K. Below *TB*, the magnetization of the ZFC curve rapidly decreases from 4.94 emu/g (at 220 K) to 1.79 emu/g (at 2 K), and the FC curve tends to be flat, which corresponds to the phenomenon that the nanocomposites show superparamagnetic behavior at 300 K.

**Figure 7:**
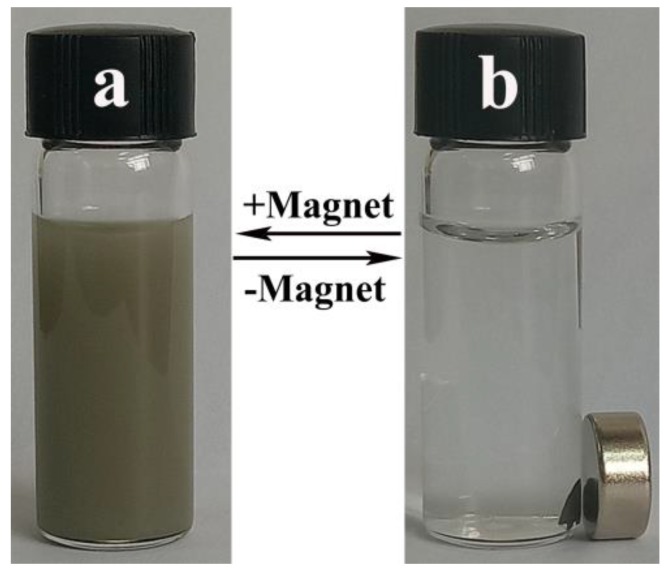
The dispersion–collection process of Fe_3_O_4_/Ag/POMs in water.

The magnetic properties of the nanocomposites facilitate their convenient and quick separation, eliminating the requirement for repeated centrifugation of the nanocomposites in practical applications. Figure 7 shows the aggregation and dispersion of nanocomposites in water. As can be seen from Figure 7, the Fe_3_O_4_/Ag/POMs nanocomposites are collected as one small piece from the uniformly distributed gray-black solution by the magnet under the influence of an applied magnetic field. The collected nanocomposites can be re-dispersed uniformly in the solvent and the process is repeatable. All the prepared nanocomposites could be collected by magnets without residual visible particles, ruling out the possibility of the existence of Ag and POMs alone, further confirming the formation of Fe_3_O_4_/Ag/POMs nanocomposites.

### 3.7. Dye Removal Activity of the Nanocomposites

The dye wastewater, derived from textiles, silk, and tanneries contains a large amount of organic pollutants which are highly toxic and carcinogenic, posing a serious threat to the environment and ecology [58,59,60]. Physical and chemical methods such as adsorption, advanced oxidation, and electrolysis have been used to remove dye wastewater [61]. Nevertheless, for adsorption, the problems of adsorption saturation and sorbent consumption are insurmountable [62]. In advanced oxidation and electrolysis processes, highly toxic derivatives may be produced and the operating costs are very high [60,63,64]. Therefore, the development of efficient methods is essential for the advanced treatment of dye wastewater. Taking the removal of MB as a probe, the removal effect of ternary nanocomposites on dye wastewater was investigated.

**Figure 8:**
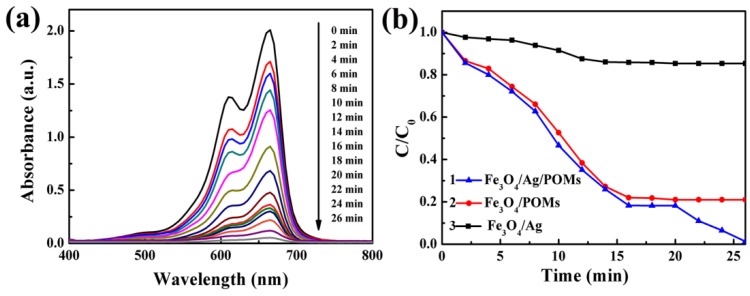
(**a**) Absorption spectra of methylene blue (MB) in the presence of Fe_3_O_4_/Ag/POMs nanocomposites, (**b**) removal of MB under irradiation of visible light after reaching adsorption equilibrium.

The removal activity of ternary Fe_3_O_4_/Ag/POMs nanocomposites was investigated by removing MB from dye wastewater. Figure 8a shows the UV–vis absorption spectra of MB with the presence of Fe_3_O_4_/Ag/POMs at different intervals. Under stirring in the dark, the intensity of the main absorption peak gradually decreases with the increase of time within 0–16 min and remains unchanged at 16–20 min to reach adsorption equilibrium, indicating the adsorption activity of Fe_3_O_4_/Ag/POMs. In 20–26 min, the intensity of the main absorption peak decreases with the increase of time under visible light irradiation, which is assigned to the photodegradation of MB. Figure 8b displays a comparative study for the removal of MB in the presence of (1) Fe_3_O_4_/Ag/POMs, (2) Fe_3_O_4_/POMs, and (3) Fe_3_O_4_/Ag under visible light irradiation after reaching adsorption equilibrium. The ordinate is represented by C/C_0_, where C is the concentration corresponding to the maximum absorption wavelength of MB at each time interval, and C_0_ is the initial concentration of MB. With the presence of Fe_3_O_4_/Ag, MB was photodegraded after reaching adsorption equilibrium revealing a removal efficiency of 14.7% (curve 3). For Fe_3_O_4_/POMs, only an adsorption efficiency of 79.0% emerged with no photodegradation of MB (curve 2). However, with the addition of Ag, Fe_3_O_4_/Ag/POMs achieved a removal efficiency of 98.7% indicating interesting photocatalytic activity besides adsorption capacity for removing dye (curve 1). The improved removal efficiency arising from nanoengineering Fe_3_O_4_, Ag, and POMs into a single entity might be attributed to the fact that Ag serves as an electron acceptor, by which chemisorbed molecular oxygen reacts with photogenerated electrons to form an active oxygen species, facilitating the trapping of photogenerated electrons, in addition to the electrostatic interactions between adsorbents and dye molecules [49]. Fe_3_O_4_/Ag/POMs nanocomposites are expected to become a novel dye remover with adsorption and photocatalysis functions at the same time, since the synergistic effect of POM and Ag can more effectively remove organic dyes from water pollution.

**Figure 9:**
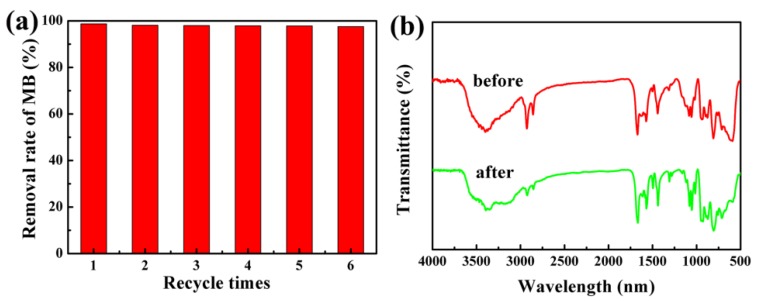
(**a**) Histogram of recycling to remove MB for six cycles and (**b**) FTIR spectra as-synthesized and after cyclic removal of MB six times with the Fe_3_O_4_/Ag/POMs nanocomposites.

For the reusability investigation, the Fe_3_O_4_/Ag/POMs nanocomposites were magnetically collected and washed with distilled water. The recovered nanocomposites were again used to remove MB under the same reaction conditions. The results showed that the removal efficiency of the nanocomposites decreased only by about 1.2% after six repeated tests (Figure 9a). The FTIR spectrum of the nanocomposites collected after the removal of methylene blue was similar to that obtained for as-synthesized, indicating the stability and resistance to light corrosion of the nanocomposites (Figure 9b). The good reusability and stability of removers are very important factors to avoid waste and reduce consumption in the actual application of dye removal.

## 4. Conclusions

In conclusion, we successfully synthesized the ternary nanocomposites Fe_3_O_4_/Ag/POMs by coating POMs on the surface of Fe_3_O_4_/Ag (core-shell) nanoparticles. The systematic characterization proves nanocomposites of core–shell–core nanostructure, narrow particle size distribution, high crystallinity, and good optical absorption properties in water. Moreover, the ternary nanocomposites Fe_3_O_4_/Ag/POMs show good superparamagnetic behavior at room temperature and the *T_B_* of nanocomposites is 220 K at a field strength of 500 Oe. This is beneficial to their applications in the fields of catalysis, biology, medicine, wasterwater treatment etc. In the experiment for removing MB, Fe_3_O_4_/Ag/POMs achieved a removal efficiency of 98.7%, revealing interesting photocatalytic activity in addition to adsorption capacity for removing dye. Simultaneously, in the cycle test, the nanocomposites had almost similar removal efficiency for MB; the FTIR spectrum of the nanocomposites collected after the removal of MB was similar to that obtained for as-synthesized, indicating stability and resistance to light corrosion of the nanocomposites. It could be proved that nanocomposites with magnetic properties, high stability, and recyclability have the potential to be novel magnetic dye removers.

## Supplementary Materials

The following are available online at https://www.mdpi.com/2079-4991/9/9/1255/s1, Figure S1: SEM-EDS analyses of Fe_3_O_4_/Ag nanoparticles. Figure S2: Thermogravimetric analyses of (a) Fe_3_O_4_/Ag nanoparticles and (b) Fe_3_O_4_/Ag/POMs nanocomposites. Figure S3: (a) STEM image and (b–i) corresponding elemental mappings of Fe_3_O_4_/Ag/POMs nanocomposites.
