# Supplementary material for: Synthesis, Characterization and Dye Removal Behavior of Core–Shell–Shell Fe3O4/Ag/Polyoxometalates Ternary Nanocomposites
Source: Nanomaterials (Basel). 2019 Sep 4;9(9):1255. doi: 10.3390/nano9091255 (PMC6780926; doi:10.3390/nano9091255)
Supplement: Supplementary file 1 [file nanomaterials-09-01255-s001.pdf]

nanomaterials

Article

Synthesis, Characterization and Dye Removal Behavior of Core–Shell–Core Fe_3O_4/Ag/Polyoxometalates Ternary Nanocomposites

Shixia Zhan, Chunyan Li, Heyun Tian, Chenguang Ma, Hongling Liu *, Jie Luo * and Mingxue Li *

Henan Key Laboratory of Polyoxometalates, Institute of Molecular and Crystal Engineering, College of Chemistry and Chemical Engineering, Henan University, Kaifeng 475004, China

* Correspondence:Correspondence: hlliu@henu.edu.cn (H.L.), luojie@henu.edu.cn (J.L.), limingxue@henu.edu.cn (M.L.)

Received: 09 August 2019; Accepted: 01 September 2019; Published: date

The elemental content of the Fe_3O_4/Ag nanoparticles was tested by SEM-EDS analysis in Figure S1. According to Figure S1, the Ag element content is 44.21% and the Fe element content is 40.13% in Fe_3O_4/Ag nanoparticles.

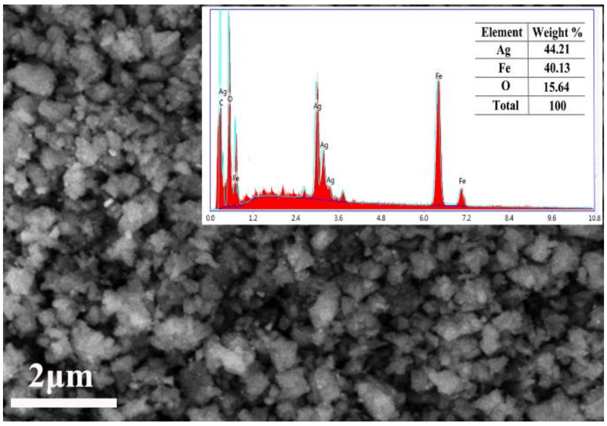

Figure 1. SEM-EDS analyses of Fe_3O_4/Ag nanoparticles.

Thermogravimetric analyses of Fe_3O_4/Ag nanoparticles and Fe_3O_4/Ag/POMs nanocomposites were performed under a nitrogen flow (Figure S2(a,b)). Figure S2 (a) shows the TG curve for Fe_3O_4/Ag nanoparticles. It can be seen from the Figure S2 (a) in the figure that the weight loss of Fe_3O_4/Ag nanoparticles can be divided into two stages. The weight loss of 4.6% from room temperature to 260 °C is mainly due to the removal of physically adsorbed water and chemisorbed water. The weight loss in the second stage is 11.92%, corresponding to the volatilization and decomposition of PEO-PPO-PEO molecules coated on the surface of the nanoparticles (between 260 °C and 450 °C). Figure S2 (b) shows the TG curve of Fe_3O_4/Ag/POMs nanocomposites. It can be seen from the Figure S2 (b) that the weight loss of the Fe_3O_4/Ag/POMs nanocomposites can be divided into three stages. The weight loss of 4.1% from room temperature to 260 °C is mainly due to the removal of physically adsorbed water and chemisorbed water. The weight loss in the second stage at 260–400 °C is 11.46%, corresponding to the fracture of the organic ligand of POMs, and the third stage 400–730 °C is the frame collapse stage of POMs with a weight loss of 28.1%. It turns out that the weight ratio of POMs in the synthesized nanocomposites is 83.5%.

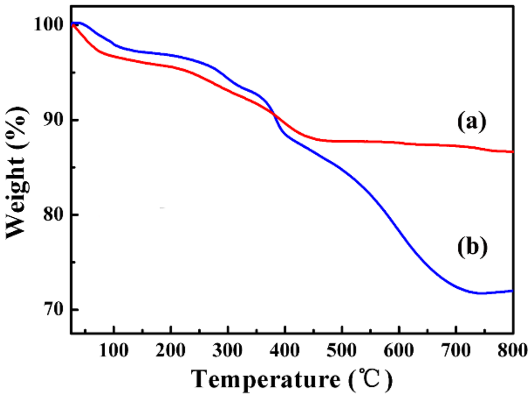

Figure 2. Thermogravimetric analyses of (**a**) Fe_3O_4/Ag nanoparticles and (**b**) Fe_3O_4/Ag/POMs nanocomposites.

The elemental mappings (Figure S3 (a–i)) illustrate the distribution of the elements Ag, Fe, Mo, P, Cu, C, O and N in the Fe_3O_4/Ag/POMs nanocomposites, indicating that Ag, Fe_3O_4 and POMs coexist in Fe_3O_4/Ag/POMs nanocomposites. These further confirm the successful formation of Fe_3O_4/Ag/POMs nanocomposites.

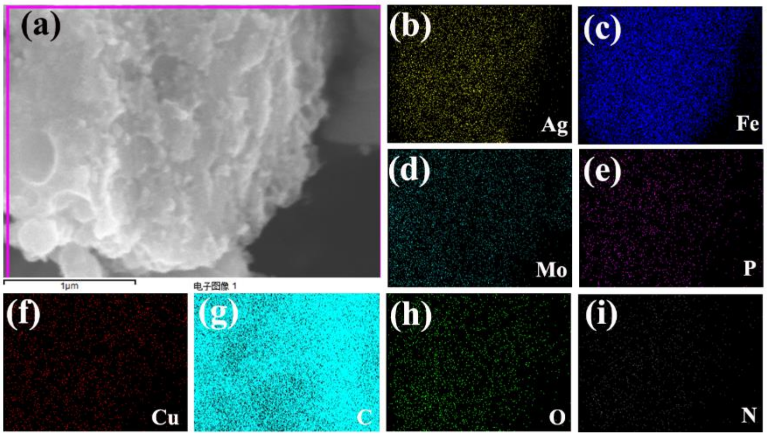

Figure 3. (**a**) STEM image and (**b–i**) corresponding elemental mappings of Fe_3O_4/Ag/POMs nanocomposites.

© 2019 by the authors. Submitted for possible open access publication under the terms and conditions of the Creative Commons Attribution (CC BY) license (http://creativecommons.org/licenses/by/4.0/).
